# Ethical and practical considerations for cell and gene therapy toward an HIV cure: findings from a qualitative in-depth interview study in the United States

**DOI:** 10.1186/s12910-022-00780-1

**Published:** 2022-04-09

**Authors:** Karine Dubé, John Kanazawa, Hursch Patel, Michael Louella, Laurie Sylla, Jeff Sheehy, Lynda Dee, Jeff Taylor, Jen Adair, Kim Anthony-Gonda, Boro Dropulić, John A. Sauceda, Michael J. Peluso, Steven G. Deeks, Jane Simoni

**Affiliations:** 1 Gillings School of Global Public Health, University of North Carolina Chapel Hill, 4108 McGavran-Greenberg Hall, Chapel Hill, NC 27599 USA; 2 defeatHIV Collaboratory, 1100 Fairview Avenue North, E5-110, Seattle, WA 98109 USA; 3 Independent Consultant, 1999 Harrison Street, Suite 1650, Oakland, CA 94612 USA; 4 AIDS Action Baltimore, 14 East Eager Street, Baltimore, MD 21202 USA; 5 Delaney AIDS Research Enterprise (DARE) Community Advisory Board (CAB), 995 Potrero Avenue, San Francisco, CA 94110 USA; 6 HIV + Aging Research Project – Palm Springs (HARP-PS), 1775 East Palm Canyon Drive, Suite 110-349, Palm Springs, CA 92264 USA; 7 Fred Hutchinson Cancer Research Center, 1100 Fairview Avenue N, Seattle, WA USA; 8 Division of Medical Oncology, Department of Laboratory Medicine and Pathology, University of Washington, 825 Eastlake Ave E, Seattle, WA USA; 9 Caring Cross, 708 Quince Orchard Road, Suite 250D, Gaithersburg, MD USA; 10 Department of Medicine, Division of Prevention Science, Center for AIDS Prevention Studies (CAPS), University of California, San Francisco (UCSF), 550 16th Street, 3rd Floor, San Francisco, CA 94158 USA; 11 Department of Medicine, Division of HIV, Infectious Diseases, and Global Medicine, University of California, San Francisco (UCSF), San Francisco General Hospital, Ward 84, Building 80, San Francisco, CA 94110 USA; 12 Departments of Psychology and Global Health, University of Washington, 3909 Stevens Way CE, Box 351525, Seattle, WA USA; 13 UNC Gillings School of Global Public Health, 4108 McGavran-Greenberg Hall, Chapel Hill, NC 27516 USA

**Keywords:** HIV, HIV cure research, Cell and gene therapy, Empirical ethics research, People living with HIV

## Abstract

**Background:**

HIV cure research involving cell and gene therapy has intensified in recent years. There is a growing need to identify ethical standards and safeguards to ensure cell and gene therapy (CGT) HIV cure research remains valued and acceptable to as many stakeholders as possible as it advances on a global scale.

**Methods:**

To elicit preliminary ethical and practical considerations to guide CGT HIV cure research, we implemented a qualitative, in-depth interview study with three key stakeholder groups in the United States: (1) biomedical HIV cure researchers, (2) bioethicists, and (3) community stakeholders. Interviews permitted evaluation of informants’ perspectives on how CGT HIV cure research should ethically occur, and were transcribed verbatim. We applied conventional content analysis focused on inductive reasoning to analyze the rich qualitative data and derive key ethical and practical considerations related to CGT towards an HIV cure.

**Results:**

We interviewed 13 biomedical researchers, 5 community members, and 1 bioethicist. Informants generated considerations related to: perceived benefits of CGT towards an HIV cure, perceived risks, considerations necessary to ensure an acceptable benefit/risk balance, CGT strategies considered unacceptable, additional ethical considerations, and considerations for first-in-human CGT HIV cure trials. Informants also proposed important safeguards to developing CGT approaches towards an HIV cure, such as the importance of mitigating off-target effects, mitigating risks associated with long-term duration of CGT interventions, and mitigating risks of immune overreactions.

**Conclusion:**

Our study identified preliminary considerations for CGT-based HIV cure across three key stakeholder groups. Respondents identified an ideal cure strategy as one which would durably control HIV infection, protect the individual from re-acquisition, and eliminate transmission to others. Known and unknown risks should be anticipated and perceived as learning opportunities to preserve and honor the altruism of participants. Preclinical studies should support these considerations and be transparently reviewed by regulatory experts and peers prior to first-in-human studies. To protect the public trust in CGT HIV cure research, ethical and practical considerations should be periodically revisited and updated as the science continues to evolve. Additional ethics studies are required to expand stakeholder participation to include traditionally marginalized groups and clinical care providers.

**Supplementary Information:**

The online version contains supplementary material available at 10.1186/s12910-022-00780-1.

## Background

Human immunodeficiency virus (HIV) cure-related research involving cell and gene therapy (CGT) has intensified in recent years [1]. Such efforts aim to either completely eliminate HIV from the body or confer sustained drug-free viral suppression [2–4] (hereafter referred to as HIV cure research). Fundamentally, cell therapies involve delivering living cells inside the human body, while gene therapies involve delivering genetic material [5]. Timothy Ray Brown—the Berlin patient—was the first person cured of HIV following a risky allogeneic stem cell transplant from a donor with a double Δ32 mutation in the C–C Chemokine Receptor Type 5 (CCR5) gene [6, 7]. His HIV cure represented a monumental scientific breakthrough that was only replicated a decade later when Adam Castillejo—the London patient—underwent a similar procedure [8–10]. Timothy and Adam’s cures have energized the HIV cure research field. In 2022, another HIV cure was potentially achieved in a woman using haplo-cord transplant from a donor homozygous for the Δ32 mutation gene deletion [11]. Several investigators are trying to replicate these cures using CGT as a means of avoiding risky transplants. To date, over 35 clinical trials involving CGT to achieve an HIV cure have been completed [12].

The field of CGT is fast-moving and raises several ethical and practical concerns. A cautionary tale remains the 1996 X-linked severe combined immunodeficiency (SCID) clinical trial in Europe, after which 25% of participants developed T cell acute lymphoblastic leukemia due to vector insertion near a proto-oncogene [13, 14]. Three years later, in the United States, Jesse Gelsinger, a volunteer in a gene therapy clinical trial for a rare metabolic disorder who had until then survived through dietary restrictions and medical therapy, died from an intense inflammatory response that led to systemic organ failure. This tragedy led to a close examination of the pace of clinical investigation in the field and substantially delayed many research efforts [15]. The first generation of CGT HIV cure interventions presents many uncertainties, toxicities, and participant burdens. Even more ethical concerns are raised when, in the event of CGT, germline cells are modified in order to potentially cure HIV. In 2018, He Jiankui announced he used clustered regularly interspaced short palindromic repeats (CRISPR) to create the first gene-edited babies in an attempt to prevent their HIV infection [16, 17]. This was widely considered an ethical failure and the effort received widespread condemnation [18]. Jiankui and his collaborators also received prison sentences and fines for these experiments.

Considering these paradigmatic cases, the field of CGT has made much progress in safety and the responsible conduct of research over the last two decades. Likewise, the regulatory environment has evolved to accommodate a recent surge in CGT research globally [14]. Since 2017, the CGT field has produced several U.S. Food and Drug Administration (FDA) approved therapeutics in multiple disease areas, such as leukemia and lymphoma, among others [14, 19]. Investigational CGT HIV cure strategies encompass several different approaches that aim to make cells resistant to HIV infection, increase immunity, or disarm HIV [20]. Common gene editing platforms disrupt the CCR5 locus and make CD4+ T cells refractory to HIV. These include CRISPR, transcription activator-like effector nucleases (TALENs), and zinc finger nucleases (ZFNs), among others [1]. An increasing array of CGT approaches are derived from the oncology field, such as chimeric antigen receptor (CAR) T cells that seek to improve the adaptive immune response against HIV [21, 22]. Some studies also require the interruption of antiretroviral treatment (ART), termed analytical treatment interruption (ATI) to determine the effect of the intervention on the immune system.

There are a growing number of CGT HIV cure clinical trials being implemented globally. While CGT will continue to present challenges in terms of efficacy and human safety, a growing toolbox of recent and future scientific advances provide cause for optimism [5, 21]. As CGT HIV cure research intensifies, the application of CGT to scientific endeavors aimed at finding a cure may heighten ethical complexities [23].

The present study builds on a growing literature in the field of HIV cure research ethics in the U.S. and internationally; however, to date, this literature has not focused on CGT HIV cure research. In 2013, Lo and Grady offered eight normative ethical points to consider for ethical HIV cure research, such as collaborative partnerships, social value, scientific validity, fair selection of participants and study sites, favorable benefit/risk balance, independent and ethical review, informed and voluntary consent, and respect for patients and communities [24]. Sugarman later called for an expanded view of ethics as well as comprehensive approaches to protecting participants [25]. Subsequent reviews have endorsed procedures to mitigate and offset risks to trial participants and increase social value of research [26, 27]. Four areas of normative and empirical HIV cure research ethics have received marked attention, including acceptable benefits and risks [28–33], the ethics of informed consent [34–36], the ethics of ATIs [37–41], and the ethics of mitigating risks to partners [42–46]. Scholars have recently called for more detailed inquiry into the ethical considerations for specific types of HIV cure research strategies that show promise, such as CGT [47, 48]. A growing number of articles, mostly from the U.S.-based literature, propose joint ethical and practical points to consider during HIV cure trial implementation [45, 49–51].

Our primary research question therefore aims to identify preliminary ethical and practical considerations to guide future implementation of CGT HIV cure research. To elicit these considerations, we implemented a qualitative, in-depth interview study with three key stakeholder groups in the United States: (1) biomedical CGT HIV cure researchers, (2) bioethicists, and (3) community stakeholders. We conducted in-depth interviews to identify preliminary ethical and practical considerations to guide future implementation of CGT HIV cure or cure-related research (both expressions were used during the interviews) [52, 53]. We chose in-depth interviews to qualitatively capture and describe nuanced ethical considerations [54, 55] from informants. Normative ethics, or how one *should* morally act, can be contextualized by empirical research ethics methods, which permit evaluation of stakeholders’ thoughts about what should ethically occur in the real-world—in this case, CGT HIV cure research. Our ultimate objective was to understand stakeholder perspectives around CGT and generate ideas on possible safeguards to help ensure CGT HIV cure research remains ethically acceptable to as many stakeholders as possible as it advances.

## Methods

### Qualitative interviews

Qualitative interviews were chosen to initiate conversations around the topic of ethical CGT HIV cure research. We wanted to establish a preliminary set of concerns and considerations [56] of different stakeholder groups in the United States based on a small sample.

### Participant selection

We used purposeful sampling to select key informants. We recruited biomedical researchers actively working in the field of CGT towards an HIV cure, community members, including people living with HIV (PLWH) who have previously participated in CGT research, as well as community advocates working in the field of HIV cure research, and bioethicists. An External Advisory Group listed potential informants who represented academic institutions, community advisory boards (CABs), government, and the pharmaceutical industry. All participants were recruited based on prior familiarity with CGT towards an HIV cure. The study’s principal investigator (K.D.) sent formal e-mail invitations to potential informants. Email correspondence indicated the purpose of the study and contained our institutional review board (IRB)-approved informed consent document, blank demographic sheet, and a sample interview guide. Potential informants self-nominated for study participation by accepting the interview invitation. All individuals who accepted the interview invitation received a Health Insurance Portability and Accountability Act (HIPAA)-compliant virtual conferencing weblink over which the interview was conducted. No self-nominating participants were excluded from the study.

At the outset, we want to acknowledge the methodological limitations of our study. Our sample size was small and directed towards stakeholders with prior involvement in CGT for HIV cure-related research. From within this group, participants self-selected. Our sample may have been biased towards individuals supportive of CGT strategies towards an HIV cure. Further, we acknowledge that our sample lacked diversity with respect to race and ethnicity and sex and gender, as most informants were white males; this is a major limitation of our study. After 19 interviews, with a significant bias towards biomedical researchers and only one bioethicist, it is likely that we did not reach saturation, the point when no new information emerges [57]; our study was designed as formative research. We did not delve into ethical considerations related to interrupting HIV treatment, as these are thoroughly reviewed elsewhere [38, 39, 42, 45, 58]. Our research was not designed as a consensus study. Despite these limitations, our study generated preliminary considerations to guide the blossoming research of CGT towards an HIV cure.

### Data collection

Two trained researchers (K.D. and J.K.) conducted all interviews in English with fidelity to the IRB-approved interview guide (Table 1). Interviews lasted between 30–60 min, and all informants agreed to be audio recorded. For equity reasons, community members received compensation in the form of an electronic US $20 e-gift card following their interviews. Informants from academic institutions, government, and pharmaceutical companies did not receive compensation as they were employed in salaried positions at the time of study participation. For government-employed informants, compensation is not permitted. For academic and pharmaceutical company employees actively working in the CGT HIV cure field, compensation would be considered a conflict of interest.

**Table 1:** IRB-approved interview guide: ethical and practical considerations for cell and gene therapy towards an HIV cure

*Interview guide* Can you please describe your involvement in HIV-related research? *Perceptions of CGT and benefit/risk considerations* What might be some of the benefits of cell and gene therapy approaches towards an HIV cure? What might be some of the risks of cell and gene therapy approaches towards an HIV cure? How do we ensure cell and gene therapy HIV cure research approaches remain within acceptable benefit/risk parameters? Are there cell and gene therapy strategies that you would consider too risky or unacceptable for human testing? Can you please explain? What are some additional ethical considerations for developing cell and gene therapy approaches towards an HIV cure? What ethical criteria should be used specifically when evaluating first-in-human (FIH) cell and gene therapy HIV cure research protocols? How do we determine when we have enough pre-clinical evidence to move cell and gene therapies into human studies? *Safeguards and risk mitigation strategies* What general safeguards should be in place when developing cell and gene therapy approaches? What safeguards should be in place when combining cell and gene therapy approaches? What are some of the ways to mitigate risks when developing cell and gene therapy approaches? What are some of the ways we can prevent off-target effects? What are some of the ways we can control the duration of the intervention? What are some of the ways we can prevent potential immune over-reaction? What are some of the other ways to prevent adverse effects?	

### Data analysis

Interviews were transcribed by a professional, web-based transcription company. One research team member (J.K.) reviewed all transcripts for accuracy against the audio recordings. We then destroyed audio recordings after cross-checking transcripts for quality. Because this was a formative research project, we employed conventional content analysis focused on inductive reasoning to analyze the qualitative data [52]. We distilled the interview data to derive key themes and generate ethical and practical considerations related to CGT towards an HIV cure.

A research team member (J.K.) compiled all de-identified responses into a single master document for manual coding. To realize the full potential of the qualitative data, the research team analyzed the data by question blocks. We carefully reviewed responses to each question to re-familiarize ourselves with the data. We then extracted salient quotes and ascribed themes and codes. Our resultant codebook was inductive and contained code names, code descriptions, and examples. Research team members (K.D. and J.K.) double-coded the data and organized text segments into emergent themes. During the coding process, we expanded and reduced codes and themes as needed. We resolved discrepancies by discussion and consensus during virtual meetings. The lead author (K.D.) summarized the key themes and wrote narrative summaries to contextualize the data. All co-authors reviewed the data.

### Ethics statement

This study received IRB approval from the University of North Carolina at Chapel Hill (UNC-CH) IRB (study #19-0522). All informants provided verbal consent to be interviewed.

## Results

We invited 38 potential informants, of whom 19 agreed to be interviewed (50% response rate). We interviewed 13 biomedical researchers, 5 community members, and 1 bioethicist. These included 16 cisgender men and 3 cisgender women. Of these, 16 were White/Caucasian, 1 was Black/African American, 1 was mixed race, and 1 was Asian (Table 2). Interviewees worked in the field of HIV for a mean of 24.1 years (SD = 10.5 years), and in HIV cure research for a mean of 14.3 years (SD = 9.7 years).

**Table 2:** Demographic characteristics of key informant interview participants (United States, 2020–2021)

Participant number	Sex	Race/ethnicity	Informant type
01	Male	White/Caucasian	Community member
02	Male	White/Caucasian	Researcher
03	Male	White/Caucasian	Researcher
04	Male	White/Caucasian	Researcher*
05	Male	White/Caucasian	Community member
06	Male	White/Caucasian	Researcher
07	Female	White/Caucasian	Researcher
08	Male	White/Caucasian	Community member
09	Male	White/Caucasian	Researcher
10	Male	White/Caucasian	Researcher
11	Male	White/Caucasian	Researcher
12	Male	White/Caucasian	Researcher*
13	Male	Black/African American	Community member
14	Male	White/Caucasian	Bioethicist
15	Male	White/Caucasian	Researcher
16	Male	White/Caucasian	Community member
17	Female	Other, Mixed Race	Researcher*
18	Male	White/Caucasian	Researcher*
19	Male	Asian	Researcher

*Biomedical researchers who work in the pharmaceutical industry

The main findings of this study relate to the views expressed by the study participants regarding: (1) Perceptions of CGT and Benefit/Risk Considerations, and (2) Safeguards and Risk Mitigation Strategies. Preliminary ethical and practical considerations for cell and gene therapy towards an HIV cure have been summarized in Table 3. Select quotes can be found in the main text. Additional file 1: Table S1 contains additional quotes.

**Table 3:** Preliminary ethical and practical considerations for cell and gene therapy towards an HIV cure

1. Perceptions of CGT and benefit/risk considerations	
1.1 Perceived benefits of CGT towards an HIV cure	Research teams should maximize the possible clinical and scientific benefits of CGT approaches towards an HIV cure. Perceived benefits included the prospect of developing “single-shot” regimens that could be less burdensome (although CGT may not prevent against re-infection), as well as scientific advancements that could lead to curative innovations for other molecular genetic diseases
1.2 Perceived risks of CGT towards an HIV cure	Research teams should minimize the possible clinical and non-clinical risks of CGT approaches towards an HIV cure. The possibility of unknown clinical risks will require careful and sustained pharmacovigilance. The risks of unintentional HIV transmission to sexual partners, therapeutic or curative misconceptions, and financial burdens of CGT should be minimized as well. Research teams should attempt to minimize social and economic risks of CGT trials
1.3 Ensuring acceptable benefit/risk balance	To ensure acceptable benefit/risk parameters, research teams should use an incremental scientific approach, ensure adequate regulatory review, minimize risks as much as possible, be transparent about potential risks, collect as much safety and efficacy data as possible, and maximize possible long-term benefits to humanity (knowledge/risk calculus) [97]
1.4 CGT strategies perceived to be unacceptable for human testing	There appears to be convergence on the unethicality of editing the germline and conducting allogeneic stem cell transplants in otherwise healthy PLWH. Research teams should remain attuned to unacceptable risk thresholds for individual study participants
1.5 Additional ethical considerations for CGT approaches towards an HIV cure	Additional ethical considerations for developing CGT HIV cure research approaches—although not unique to the field of CGT—include strong scientific rationale, fair participant selection, robust informed consent, distributive justice, and equity issues. Research teams should carefully inform trial participants about what adverse events to look for following a CGT intervention. CGT researchers should try to maximize long-term benefits for the HIV community
1.6 Considerations for first-in-humans CGT HIV cure trials	Considerations for implementing FIH CGT HIV cure trials—although not specific to this field—include a compelling scientific rationale for moving into human testing, robust pre-clinical data despite limitations of current animal models, close observance of the regulatory process, and involvement of PLWH in trial design. For a comprehensive FDA summary regarding *Preclinical Assessment of Investigational Cellular and Gene Therapy Products*, see https://www.fda.gov/media/87564/download
2. Safeguards and risk mitigation strategies	
2.1 General safeguards for developing CGT approaches towards an HIV cure	Safeguards to developing and implementing CGT approaches towards an HIV cure may include, but are not limited to, clinical trial design considerations for example, narrow inclusion and exclusion criteria, low initial trial enrollment, dose escalation and de-escalation rules, staggering trial participants, careful monitoring for potential side effects that include long-term side effects, and clear stopping rules in the event of intolerable toxicity. CGT product specificity, manufacturing and transport safeguards (e.g., to ensure identity, purity, sterility, stability, and potency), robust research staff training, accumulating a scientific body of evidence over time, and monitoring for potential conflicts of interest of investigators are also of paramount importance
2.2 Safeguards for combining CGT approaches	Possible safeguards for combining CGT approaches may include but are not limited to, ensuring individual components are safe, determining potential harmful combinatorial or synergistic effects, combining existing safeguards, continued investment in pre-clinical work, ensuring favorable benefit/risk profiles, transparency about risks, and community involvement. See FDA Combination Products Guidance Documents, available at: https://www.fda.gov/regulatory-information/search-fda-guidance-documents/combination-products-guidance-documents
2.3 Mitigating off-target effects of CGT interventions	Possible risk mitigation strategies for off-target effects may include but are not limited to, improved targeting during engineering, extensive testing for off-target effects, such as location of off-targeting, risks of off-targeting, and frequency of off-targeting, careful monitoring in the entire body, including both blood and tissue sampling, ensuring that trial participants clearly understand possible long-term risks so they know what to look for over time, and long-term participant follow-up. The risk of double stranded breaks should be minimized as much as possible. See FDA Guidance on *Long-Term Follow-Up After Administration of Human Gene Therapy Products*, available at: https://www.fda.gov/regulatory-information/search-fda-guidance-documents/long-term-follow-after-administration-human-gene-therapy-products
2.4 Mitigating risks associated with long-term duration of CGT interventions and immune over-reactions	Possible risk mitigation strategies to control the long-term duration of CGT interventions depend on the strategies being investigated, for example, gene editing may warrant transient approaches while immune-based approaches may warrant more frequent monitoring and control. Carefully designed strategies to control the durability of a CGT investigational product, such as genetic manipulation, safety switches or ART restart should be implemented Possible risk mitigation strategies for immune overreactions, also called cytokine release syndrome, a risk factor associated with some CGT interventions (such as CAR-T cells) include active monitoring, using the ASBMT consensus grading system and established pharmacological protocols to reduce inflammation, like cortical steroids. Possible risks of neurotoxicity should also be monitored and carefully mitigated

1. Perceptions of CGT and Benefit/Risk Considerations

Interview topics included: (1) perceived benefits of CGT towards an HIV cure, (2) perceived risks, (3) considerations necessary to ensure an acceptable benefit/risk balance, (4) CGT strategies considered unacceptable, (5) additional ethical considerations, and (6) considerations for first-in-human (FIH) CGT HIV cure trials.

1.1. Perceived Benefits of CGT Towards an HIV Cure

Perceived benefits included the greater likelihood of achieving a complete cure when compared with other HIV cure strategies under investigation and the prospect of developing “one-shot” cures that could be globally scalable.

The bioethicist (#14) stated CGT approaches that aim at being “one-and-done” therapies could be more attractive to PLWH because they would not require lifelong treatment if proven effective, although this would not be true if PLWH would not be protected against HIV re-infection.

*I think the distinction is between one and done therapies… that are really like, "Okay, we do this intervention and, once we're through it, you're done. You don't have to worry about your HIV anymore… You don't have to continue to take antiretrovirals" versus something where we're talking about continued lifelong therapies… I can imagine that if there were something that could be one-and-done, and that were reasonably safe, there would be a lot of people who would be very interested in doing it for themselves.—*Bioethicist (#14)

Community members also identified possible benefits of CGT research towards an HIV cure. One community member (#16) believed CGT will be required to achieve a cure for HIV. If effective, CGT may result in making cells resistant to HIV, thereby preventing future HIV infection (#08). Another community member (#05) believed CGT strategies could go to the root of the problem to fix what is broken.

Two community members (#05, #16) described possible clinical benefits that may emerge from clinical trials even when the CGT intervention does not result in HIV cure. These community members both witnessed unexpected, significant increases in their CD4+ T cells following a non-curative CGT intervention which did not occur after a long period on salvage HIV treatment regimens.

*It's been 10 years since I was in the [CGT] trial. A secondary side effect, or secondary outcome of the trial, which was that my T-cells were doubled. I mean, nobody knew that was going to happen. So, I mean that's something that we need to consider, is what are those secondary outcomes that may come out of a gene therapy trial, that we would not expect it.—*Community member (#05)

*It might be a great adjunctive benefit if you don't get a cure. I know people that were, myself included, basically on salvage regimens and at the end of their treatment rope with resistance… [who] have had perhaps an unintended benefit that they do better than they had… before [while] on medications.—*Community member (#16)

Biomedical researchers discussed the need for cutting-edge approaches to curing HIV, since other strategies under investigation have not yet proven effective at keeping HIV suppressed without ART. Two biomedical researchers (#02, #09) stated CGT has great potential to lead to complete elimination of HIV. They described the strong scientific rationale for attempting to replace the immune system with cells that would become resistant to HIV.

Finally, a biomedical researcher (#06) described how scientific advancements in CGT could lead to cures for other molecular genetic diseases.

1.2. Perceived Risks of CGT Towards an HIV Cure

Community members and biomedical researchers described possible risks of CGT HIV cure research. Community members were concerned about both short-term and long-term clinical risks of CGT. They mentioned off-target editing and risks of developing cancer later in life as most salient to them.

Besides clinical risks, community members’ narratives centered around the risk of creating false hopes and expectations in the community.

*I think when gene therapy reaches the level of being able to cure cancers, to being able to cure HIV, it's going to be seen like a miracle. But it could also create false expectations. And I think the process of getting to that day, that's a risk, creating these false impressions. 'Cause even when I talk that way… it's easy to create false hopes and false impressions, and I think that causes more damage… over time, which may be collaterally worse than the actual cure you get to in the end.—*Community member (#08)

Likewise, biomedical researchers also described both short-term and long-term clinical risks of CGT. Immune reactions, such as cytokine release syndrome (CRS) expected with some CAR-T cell interventions, were perceived as a real possibility. Three biomedical researchers (#02, #11, #12) mentioned the risk of developing later malignancies. A biomedical researcher (#19) mentioned the theoretical risk of insertional oncogenesis, a deoxyribonucleic acid (DNA) mutation that could lead to cancer. Insertional oncogenesis may occur when a vector integrates (“inserts”) into a chromosome near an oncogene whose expression is normally repressed.

Biomedical researchers and community members were also concerned about possible unknown risks of CGT. These unknown risks will require careful vigilance, particularly as there may be inter-participant variations.

*I think the risks are the ones… that we don't know about... Any time you do something that's unknown, you can't quantify or qualify risks. There's no crystal ball… It's only going to be after many years of accumulated experience that we really know. Likely, it's going to be very heterogeneous with respect to the risks, in terms of interindividual variation. We just have to walk forward, like anything else we do in the clinic, with our eyes wide open.—*Biomedical researcher (#03)

One biomedical researcher (#09) was concerned about the risk of transmitting HIV to sexual partners during ATIs and unsuspected viral rebounds. Another biomedical research (#06) was concerned about the possible economic/financial risks of CGT.

1.3. Ensuring acceptable benefit/risk balance

Informants described the difficulty of objectively assessing benefit/risk ratios and recommended minimizing risks to participants while maximizing possible benefits to science and humanity.

The bioethicist (#14) provided three suggestions to ensure acceptable benefit/risk profiles: (1) minimizing risks as much as possible, (2) learning as much as possible from the trial, and (3) being transparent about the potential risks to allow participants to make informed decisions.

*I think here the question is really, have we minimized the risk? Is it really, really good science? Are we being honest with people so they can make informed decisions?*—Bioethicist (#14)

A community member (#08) described the difficulty of assessing benefit/risk ratios in early-phase CGT trials, comparing the exercise to “walking a tightrope.” Further, risk toleration is greatly reduced because of highly effective ART. Constant consideration around acceptable benefit/risk ratios is therefore required.

*I don't know if you ever really do achieve that… So I always think of it as walking a balance bar or walking a tightrope… [In] the case [of] cure research…, there's gonna [be] less acceptable risks… And so that's stuff that we're gonna have to constantly monitor… Don’t assume that you have a risk-benefit ratio, and it's taken care of… it needs constant vigilance.—*Community member (#08)

Biomedical researchers converged on the difficulty of making benefit/risk assessments. They described how these evaluations rely on their biological intuition based on the specific mechanisms of action being investigated. They also commented that the field of CGT HIV cure research will need better biomarkers or metrics to assess benefits and risks.

*[I]t is very difficult to really, fully assess a cost benefit ratio to make decisions like that. I think a lot of it has to [do] more with biological intuition, in terms of, based on the underlying mechanism of action associated with a curative approach, we can surmise that… it's not likely to be harmful… But I think until we have better cure biomarkers, it's going to be difficult to come up with really robust metrics to make yea or nay decisions about going to the clinic.—*Biomedical researcher (#19)

To ensure acceptable benefit/risk profiles, biomedical researchers recommended collecting as much safety and efficacy data as possible, particularly at the pre-clinical stage. They also stressed the need for an incremental scientific approach, adequate regulatory reviews, and robust risk mitigation strategies as part of clinical protocols. Researchers will also need to consider potential benefits to otherwise healthy PLWH compared with standard ART.

In addition to weighing the possible benefits to PLWH, a biomedical researcher (#09) considered the potential benefits of a curative CGT strategy to humanity if proven effective (social value).

1.4. CGT Strategies Perceived to be Unacceptable for Human Testing

There was a convergence of opinions regarding two CGT HIV cure strategies considered to pose too much risk and to be unacceptable for human testing: germline editing and allogeneic stem cell transplants in otherwise healthy volunteers.

First, informants identified gene modifications that would affect the germline to treat or cure HIV as unethical. They referenced the recent episode of embryos being gene edited using CRISPR-Cas9 by He Jiankui in China.

*I think that needs to be a very solid moratorium [on editing the germline in HIV], even though it's been broken… I'm against the germline therapy, simply because of the implications that we are not even yet aware of, and I think it's jumping the gun... I do not think we are ready to start messing with our germline.—*Community member (#08)

*Germline editing [towards an HIV cure]… at this point, I think should be a no no… Definitely with the tools and the understanding that we have now, we shouldn't even think about doing germline editing and the stuff that [He Jiankui] did with the CCR5 editing with the babies. [It is] totally irresponsible and insane.—*Biomedical researcher (#19)

Informants also converged around the unethicality of conducting allogeneic stem cell transplants in otherwise healthy PLWH, unless there was an underlying malignancy warranting such a risky procedure.

*Except for people with cancer, you wouldn't want to do a bone marrow transplant on a healthy person, right? Wow, it can lead to the cure in a relatively small percentage of the people that get it, there the risk reward doesn't make sense because there's a 30-40% chance you can die. That's never a good risk reward benefit… It's an area where we know much less because there are no proven stem cell therapies.—*Biomedical researcher (#09)

A community member (#05) said they would not tolerate any procedure that could lead to debilitation or hastened death. In turn, a biomedical researcher (#07) and community member (#08) commented that unacceptable risks would depend on individual volunteers or scientists. Further, a biomedical researcher (#12) was adamant that science should not be restrained as long as experiments were conducted within ethical boundaries.

1.5. Additional Ethical Considerations for CGT Approaches Towards an HIV Cure

We asked informants to provide additional ethical considerations for developing CGT HIV cure research. Most of the considerations given were not unique to this field and included: strong scientific rationale, safety maximization, fair participant selection, and distributive justice. Community members stressed the need to maximize long-term scientific benefits for the HIV community.

Informants noted the need for a strong scientific rationale and hypothesis, and that only approaches that could lead to a successful durable ART-free suppression regimen should be pursued. Informants recommended maximizing safety, a responsibility that often rests directly with scientists.

*Then the main driver of all of this is the validity of the hypothesis, the rationale which is driving the program. That rationale is how we start to think about risk-benefit and ensuring that that rationale can be clearly discriminated from existing studies or published literature is absolutely key. If it can't be discriminated from those other studies, then what is the purpose of repeating it?—*Biomedical researcher (#04)

Another ethical consideration relates to fair participant selection. Informants recommended recruiting volunteers who represent populations of interest and who are diverse in age, sex and gender, and race and ethnicity.

Other ethical considerations related to distributive justice. There must be a balance between providing access to HIV treatment and prevention around the world and providing research funding dedicated to an HIV cure. Efforts should also be made to reduce the cost of CGT technologies as a matter of global access justice and equity. Further, several informants emphasized the critical importance of a robust informed consent process.

Finally, a community member (#01) discussed the ethics of CGT development. Several companies conducted initial experiments in PLWH, only to move on to other disease areas when a CGT product demonstrated some safety or proof-of-concept. This community member (#01) recommended that CGT companies show a genuine commitment to stay in the HIV space and maximize long-term community benefits for altruistic PLWH.

1.6. Considerations for First-in-Human CGT HIV Cure Trials

Informants provided considerations for implementing FIH CGT HIV cure trials which included robust pre-clinical data, well-designed and supervised FIH trials, and observance of regulatory standards.

The bioethicist (#14) described the imperative for a compelling scientific rationale for implementing FIH trials. Similarly, a biomedical researcher (#12) stressed that nothing could replace FIH trials.

*If there's a bunch of preclinical experience that can be done to narrow the window of uncertainty, then those should be done. But at a certain point… if there's nothing more than that we could do before going into humans to actually take the science forward, then you get presented with the million dollar question which is, "Are we willing to do this?" That is going to have to be a judgment of how compelling is the science [and] how compelling is the rationale to date, and weighing that against what are the risks we might be asking people to take.—*Bioethicist (#14)

A community member (#08) emphasized the need to involve PLWH in clinical trial design from the start, as well as adequate compensation for participation, including for research-related injuries.

Biomedical researchers’ narratives centered around the need for robust testing in animal models. There must be strong emphasis on safety standards together with signals of potential efficacy in humans. A community member (#08) stressed the need for careful peer review of pre-clinical data.

*Everything plays to me around safety and then, if it's safe, I think to proceed, if you've checked preclinical development, you've checked that the genes are manufacturing, it is released. It's all this. It's clean. It's ready to be infused. Highest standards. Then together this body of evidence makes it ethical.—*Biomedical researcher (#15)

Further, biomedical researchers discussed relying on regulatory authorities and processes, such as the FDA’s investigational new drug (IND) application process or their respective Institutional Biosafety Committee (IBC), for guidance, as well as Data Safety Monitoring Boards (DSMBs).

*It's like you check so many things to make sure that it's safe to proceed, it's ethical... We have [an] Institutional Biosafety Committee… reviewing that and we have the FDA that has clinical, preclinical, and CMC [Chemistry, Manufacturing and Controls] manufacturing reviewing that body of data..., so they should know when [to begin testing].—*Biomedical researcher (#15)

Nonetheless, several biomedical researchers pointed out limitations with current animal models, such as humanized mice and non-human primates, to predict future outcomes in humans. They also mentioned the need to continue improving these pre-clinical research models.

2. Safeguards and Risk Mitigation Strategies

Interview topics included: (1) general safeguards for developing CGT approaches towards an HIV cure, (2) safeguards for combining CGT approaches, (3) mitigating off-target effects, (4) mitigating risks associated with long-term duration of CGT interventions and risks of immune overreactions.

2.1. General Safeguards for Developing CGT Approaches Towards an HIV Cure

Biomedical researchers described safeguards related to the specificity of the CGT product to ensure that the intervention being tested only targets HIV. Manufacturing and transportation safeguards (e.g., to ensure identity, purity, sterility, stability, and potency) were also mentioned, but these were perceived to be straightforward.

*Probably the major safeguard that I've looked at over the last couple of years is specificity. So, if I develop this therapy, whenever it's in your body, will it only recognize the cells that it's intended to recognize? So, I would say that's relatively easy for something for HIV, because HIV infected cells, and HIV itself, have these very specific proteins that we can target, and we can develop reagents against those.—*Biomedical researcher (#17)

Several general safeguards centered around clinical trial design issues, including dose escalation and de-escalation rules, staggering trial participants, carefully monitoring trial participants for possible adverse events (e.g., off-target effects and hyper immune responses), and clear stopping rules in the event of intolerable toxicity. In addition to having narrow inclusion and exclusion criteria, a biomedical researcher (#15) recommended paying close attention to potential social and economic vulnerabilities of study participants (such as lack of medical insurance) and being careful not to exacerbate these pre-existing vulnerabilities.

*The worst fear is the fear of the dose. So, you have to pick a first dose, and that's the most frightening, most sleep-losing part of this. What is the first dose? Because we can quickly adjust that. We can quickly make changes, but the first person dosed is going to receive the dose that we thought from all of our reading, writing, calculating, everything else, that we thought was safe. So, I'll tell you that's the stomach acid producer right there, is the first dose.—*Biomedical researcher (#04)

*The inclusion criteria are significant. You want to know who is safe to treat and who is not, [and] where you are adding more risk… So I think there's certain populations [who may lack medical insurance] that are vulnerable.—*Biomedical researcher (#15)

A biomedical researcher (#04) discussed the importance of carefully training research staff on mitigating risks from novel CGT therapies. Medications should also be readily available to reverse possible complications.

Because CGT interventions may have long-term side effects, the importance of longitudinal clinical monitoring also emerged as an important safeguard. Research teams should employ mitigation strategies for worst possible outcomes.

*If one is going to do a gene therapeutic approach, [and] I'm not sure if this still holds true, but it used to be you had to monitor those patients for life. But there is that risk that, because you're manipulating the genome in some cells, that a cell that's hanging around for quite a bit of time could suddenly, or over time, go bad [or] become malignant. [You’ve] got to watch for a very, very long time with these gene therapy approaches because there could be very long-term side effects.—*Biomedical researcher (#02)

In addition, a biomedical researcher (#15) considered the cumulative body of scientific evidence as a safeguard. Another biomedical researcher (#07) discussed monitoring for potential conflicts of interest of investigators. A community member (#08) emphasized the need for ongoing community involvement across fields of research because CGT innovations may originate from other fields, for example, cancer.

2.2. Safeguards for Combining CGT Approaches

Moreover, we inquired about possible safeguards for combining CGT approaches. Several informants described how a cure for HIV will likely require a combination regimen as opposed to monotherapies.

The bioethicist (#14) perceived that a lot of CGT approaches are already used in combination. Community members (#14, #16) and one biomedical researcher (#15) also explained that a cure for HIV will likely require combining different approaches, much like combination ART for HIV treatment.

Informants included making sure that individual CGT products were safe and carefully determining potential harmful combinatorial or synergistic effects as potential safeguards for combining CGT.

Biomedical researchers discussed how combinatorial CGT approaches will require combining existing safeguards. For example, gene editing components will require measurement of off-target editing, while CAR-T cell components will require monitoring for potential toxicities.

*When we think about multiple gene therapy approaches, usually we're thinking about multiple approaches that go into the same cells. So, for instance, if we have a CAR-T cell product where we've reprogrammed those T cells to seek out and destroy infected cells, we'll often also gene edit those cells for CCR5 so that those cells don't themselves become infected. So in that regard, it's a combination, but it's also the same cell product, so we can sort of combine the safety assurances that we have. So, for the CCR5 editing, it would be measuring the rate of off-target editing, and for the CAR-T cells, it would be proving that they're not toxic, and they're able to be controlled in the blood stream, if necessary.—*Biomedical researcher (#06)

Biomedical researchers also described how CGT products may be combined with non-CGT modalities, for example, latency-reversal agents, as part of an HIV cure research regimen. Research teams would need to provide safeguards for both interventions when designing research protocols.

Biomedical researchers perceived that regulatory guidelines were robust enough with regards to combinatorial HIV cure research. They cited the FDA’s published guidance for combining investigational products. A biomedical researcher (#12) cautioned about the need for continued vigilance around favorable benefit/risk profiles of combination regimens. The types of favorable combinations may also depend on the health status of patients/participants.

Additional safeguards for combining CGT products included carefully informing participants about the possible risks of combinatorial CGT, transparency, and robust community involvement.

2.3. Mitigating Off-Target Effects of CGT Interventions

Biomedical researchers offered possible ways to mitigate the risks of off-target effects of CGT interventions. These included better targeting of the CGT product during the engineering process, extensive testing for possible off-target effects, monitoring for potential off-target effects in the entire body (including tissue sampling), and long-term follow-up of study participants.

Biomedical researchers described how CGT technologies are improving their targeting of HIV during engineering. For example, gene editing and viral vector technologies are becoming more specific. Some technologies (e.g., CAR-T cells) already have well-defined targets, and robust specificity testing is in place for these.

Biomedical researchers further discussed extensive testing for off-target effects that are required as part of research efforts. Elaborate off-target diagnostic tests are now available but may need to be standardized across protocols (#06). Further, off-target testing should investigate where the off-targeting occurred, the possible risks of off-targeting, and the frequency of off-targeting.

*For gene editing, there are a lot of assays right now that various groups are developing with greater and greater sensitivity to measure any off-target effects. I think those assays are all really great, but I think it's getting into somewhat of an overkill... I would say if I'm not able to keep up with them... [Having] a better idea of… what the best assay is and really getting to a point where we can standardize what that assay is for clinical trials is going to be very important… Different groups are going to have different gene editing protocols in clinical trials, and they'll each have their own off-target assay, and the ability to compare one to other is going to be tough.—*Biomedical researcher (#06)

A biomedical researcher (#02) mentioned the need for monitoring off-target effects in vital organs throughout the body, as well as long-term follow-up of trial participants.

*There have been chimeric antigen receptors used in cancer studies that, for some reason, targeted cardiac tissue, and that caused problems. The therapy itself had an off-target effect and that's something you have to worry about, too, so that's where your safety testing comes in*.—Biomedical researcher (#02)

Further, three biomedical researchers (#10, #12, #19) challenged the concept of off-target effects. A biomedical researcher (#10) described how every drug has off-target effects. Informants stated that it may be best to reframe the conversation in terms of benefit/risk assessments.

*Off-target is an interesting concept, because every drug has off-target effects… Sometimes we get down the rabbit hole of thinking that precision is everything, and maybe we set our standards a little too high as a result. You take Tylenol, it has off-target effects: as well as getting rid of your headache, it's doing things throughout your body… So, I feel the conversation has got a little distorted, it's got away from risk benefit, and it's obsessing with on-target and off-target.—*Biomedical researcher (#10)

Another biomedical researcher (#19) described how even 100% on-target editing may carry some risks, such as immunological consequences derived by completely removing the CCR5 receptor.

2.4. Mitigating Risks Associated with Long-Term Duration of CGT Interventions and Immune Over-Reactions

We asked biomedical researchers to describe ways to mitigate risks associated with the long-term duration of CGT interventions. They explained that the desired duration of a CGT intervention depends on the specific product being tested and that there are engineering methods to control the duration of CGT interventions.

Biomedical researchers described how the optimal duration of CGT interventions depends on the specific CGT intervention or mechanism of action being tested. For example, transient approaches may be preferred for gene editing (e.g., CRISPR-Cas9).

*It just depends on the different types of cell and gene therapy. If your gene therapy is using… gene editing machinery, [like] CRISPR-Cas [that] goes in and cuts the gene out, then that activity is and should be very transient, but it creates a lifelong effect… You don't want to be doing gene therapy to disrupt CCR5 with a gene therapy vector that will hang around for decades; you want to go in with a very transient gene therapy… Other types of therapies… you could think about using an AAV [adeno-associated virus] vector to go into a cell and be producing… an HIV entry inhibitor or something that should act as a vaccine, there you actually want to have long term production.—*Biomedical researcher (#10)

Biomedical researchers mentioned approaches to control the duration of CGT interventions (#02). One option would be to create a safety switch; however, this switch may also carry safety issues (#17), work prematurely (#09), or provide a false sense of security (#04). ART re-initiation is another way to shut down the CGT intervention by removing the HIV target (#09).

*Obviously, the longer we can keep the therapy working, the better the chance that it actually… provide[s] therapeutic benefits. The converse obviously, is that if things go awry, how can you stop the therapies? There's a variety of these suicide strategies that have been put forward that… could do that… For HIV it's probably more feasible than in cancer... For HIV, we certainly have the ability to get rid of the antigen by re-establishing ART again. That's a great tool that we have for HIV that they don't have for cancer: we can start people back on their medicine again and then the target of the cell therapy goes away, and that probably is going to go a long way to get rid of any adverse reaction we have.—*Biomedical researcher (#09)

In addition, we asked biomedical researchers for recommendations on immune overreactions resulting from CGT interventions. CRS were perceived to be important risks associated with CAR-T cell therapies. CRS can, however, be mitigated by pharmacological approaches, for example, corticosteroids or interleukin inhibitors.

Most biomedical researchers believed the risk of immune overreactions mattered a great deal when testing CGT interventions. These could lead to a lifetime of steroid use or even death (#07). A biomedical researcher (#10) recounted the pivotal Gelsinger episode.

*Jesse Gelsinger... basically died because he had a massive immune response to a high dose of adenoviral vector, and that started this escalation that couldn't be stopped. So, as we are tweaking our immune responses, I feel we always need to remain vigilant for the unexpected.—*Biomedical researcher (#10)

CRS was perceived to be a significant risk with CAR-T cells because these engineered cells are designed to turn on an immune response (#02). Biomedical researchers recommended using established grading systems to measure the intensity of immune overreactions, such as the American Society for Blood and Marrow Transplantation (ASBMT) scale to grade CRS in addition to the ASBMT Immune effector Cell-Associated Neurotoxicity Syndrome (ICANS) Consensus Grading system to grade neurotoxicity. Besides actively monitoring for CRS, additional risk mitigations included testing for antigenicity and immunogenicity of CGT interventions (#19), using pharmacological approaches to reduce inflammation (#10), and sustained vigilance (#10).

Two biomedical researchers (#04, #17) advised involving oncologists with extensive experience dealing with CAR-T cells and CRS on research teams. There must also be an infrastructure in place to deal with adverse side effects (#17). Overall, a high level of vigilance is warranted.

## Discussion

This qualitative interview study probed key informants on preliminary ethical and practical considerations for developing CGT towards an HIV cure. Our formative research advances the empirical research ethics literature on HIV cure research by providing considerations specific to CGT [47, 48]. CGT approaches may confer scientific comparative advantages compared with other HIV cure research strategies under investigation. For example, CGT HIV cure strategies address the underlying biology of HIV infection [59] and have the potential to become one-time regimens that could eventually be scaled-up globally [1, 60], although it is unclear whether they will be able to protect against HIV reinfection risk and transmissibility [61]. As indicated by informants in our study, scientific advancements in the field of CGT HIV cure research may also benefit other molecular genetic diseases. Clinical benefits short of a cure (e.g., increase in CD4+ T cells) may also emerge in PLWH.

To ensure acceptable benefit/risk balance, we found CGT research teams must maximize scientific benefits, while minimizing risks as much as possible. This finding is consistent with much prior normative ethics literature on HIV cure research [24, 28, 62]. Also consistent with previous research is the difficulty of objectively assessing benefit/risk ratios when evaluating HIV cure research protocols [63]. According to bioethicist King, this balancing of benefits and risks is complex—one that is not only scientific, but also societal [64]. Biomedical researchers in our study noted the importance of robust regulatory review of clinical trial submissions and learning as much scientific information as possible from each CGT trial, adopting an incrementalistic view of the research process.

Another important theme that emerged from our interviews was the need for transparency around potential risks of CGT approaches. Consistent with prior research, there should also be transparency regarding potential known and unknown risks of CGT to allow PLWH to make informed decisions about whether or not to participate in trials [65, 66]. Transparency—together with disclosure and discussion of uncertainty—also protects integrity of the research process [23]. Informants in our study further recommended sustained vigilance around possible unknown risks. Notably, two CGT strategies emerged as unacceptable for human testing: germline editing and allogeneic stem cell transplantations in otherwise healthy volunteers, consistent with prior published work [67–69]. Community members in our study noted that any strategy that would cause disability or hastened death would be considered unacceptable. This is consistent with prior HIV cure socio-behavioral research in the U.S. [32, 70].

Most informants in our study focused on minimizing clinical risks, although some highlighted the critical importance of paying attention to potential social and economic vulnerabilities of CGT participants, such as lack of medical insurance. Previous socio-behavioral research in the U.S. found PLWH were more aware of potential social and financial risks of HIV cure research than other stakeholder groups [70]. Research teams must be careful not to exacerbate potential social and economic vulnerabilities for PLWH. Compensation for research-related injury should also be clearly explained in the informed consent form, as emphasized by community informants in our study.

Additional ethical considerations that emerged in our study—although not unique to CGT research—included robust pre-clinical evidence to move products into human testing [21, 64, 68], strong scientific rationale for pursuing CGT approaches, fair participant selection [71, 72], robust informed consent [34–36], and nonmaleficence and protection of participants from excessive risks [73]. Most of these ethical considerations parallel those found in recent National Academies workshop proceedings on CGT, ethics, and governance [74, 75]. In addition, informants in our study raised the ethics of CGT development that includes a sustained commitment to the HIV field. This long-term commitment to the HIV community can go a long way in developing trustworthiness of CGT research towards a cure [76]. Informants further emphasized the critical importance of distributive justice [47], particularly access to underserved communities, which would entail reducing the long-term cost of CGT technologies and make them widely scalable. Increasing attention is being paid to the role of distributive justice, equity, and scalability in the field of CGT research. For example, a novel initiative called the Global Gene Therapy Initiative (GGTI) adopts an equity framework to CGT research and aims at making CGT technologies available to resource-limited parts of the world by leveraging capacity between the fields of HIV cure and sickle cell disease [77]. In the context of significant advances in CGT towards an HIV cure and other CGT fields, it will also be important to maximize social value and ensure interventions can be scalable to resource-limited settings [77, 78].

Further, our study provided insight into the scientific, participant-level, and societal challenges of developing CGT towards an HIV cure and the long-term social acceptability of relevant research [47, 79]. Informants—community members and one biomedical HIV cure researcher in particular—emphasized the need for early, sustained, and robust community consultation in clinical trial design and reviews of CGT protocols. Several scholars involved in the field of HIV cure research have similarly emphasized the critical importance of partnering with the community at all stages of the research process to advance ground-breaking science and move towards acceptable benefits/risks [80–83]. Moreover, these findings align with recommendations made in two separate systematic reviews on increasing patient acceptability of CGT research across various disease areas [13, 84]. Providing the community with opportunities to offer input and engage in meaningful dialogue around the use of CGT towards a cure for HIV should be a priority [85], not only to increase awareness about ongoing trials, but also to understand factors that affect how communities of PLWH and allies perceive such research. As indicated in prior research, acceptability of CGT will likely be tied to perceived risk levels and invasiveness of interventions [13]. In a prior study implemented by our group among racial and ethnic minority groups in the U.S. we found important misconceptions and mistrust around CGT HIV cure research [86]. For example, several PLWH believed a cure for HIV had already been achieved and was systematically being withheld from the poor, while others believed that only participants who were desperate should participate in CGT trials [86]. Given the complexity of CGT science, there will also need to be effective communication strategies for the public that simplify information about the research and its goals. Moving forward, perspectives of PLWH who participate in CGT will also be important to understand. Our study also uncovered the need to remain extremely cautious in our description of expectations for early-phase CGT trials to avoid the risk of therapeutic or curative misconception [61, 65, 66] and the risk of creating false hopes and expectations about what the science can deliver [87].

The ethics of translational research for CGT products may require heightened considerations when compared to other HIV cure research approaches, particularly with regard to their specificity, risk profiles, and irreversibility [71, 88, 89]. A strength of our paper is the identification of potential safeguards and risk mitigation strategies for developing and implementing CGT approaches. Informants carefully described considerations related to clinical trial design, research oversight, long-term monitoring of trial participants, and constant vigilance. A cure for HIV will likely require a combination of approaches, increasing potential risks above standard of care. Informants suggested determining harmful combinatorial and synergistic effects and combining safeguards. These findings align with our prior empirical research ethics work around designing ethical and safe combination HIV cure trials [49]. A major concern with CGT has been the risk of off-target effects, but highly sensitive tests are now available to detect and mitigate these effects [65, 90]. Informants in our study emphasized the need for greater standardization in how these off-target diagnostics are employed across CGT trials. Other scholars have similarly emphasized the critical importance of CGT product specificity and efficiency as critical safeguards [1, 90, 91]. Some biomedical researchers in our study recommended reframing off-target conversations in terms of benefits/risks assessments. Not mentioned by informants in our study, but still of critical importance, would be the need to minimize risks of double stranded breaks (DSB), which can lead to mutations, loss of heterozygosity and chromosome rearrangements that could result in cell death or cancer [92–96]. Finally, the desired duration of CGT interventions will depend on the specific strategy under investigation. For CAR-T cells, research teams will need to carefully monitor for CRS and neurotoxicity using established guidance and ensure the availability of countermeasures to reverse possible complications.

## Limitations

We acknowledge the methodological limitations of our study outlined above (see “Methods”). As we move forward with CGT HIV cure research, we will need to remain cognizant of dissenting opinions. Our team is implementing further research to better understand perceptions of racial, ethnic, sexual and gender minorities around CGT HIV cure research in the U.S. [86]. Our study did not address special safeguards that should be in place for pregnant women, pediatric populations, and other potentially vulnerable groups with regards to CGT towards an HIV cure, and these should be further examined. Another key stakeholder group to survey around the acceptability of CGT towards an HIV cure would be care providers. Additional stakeholder engagement will be necessary to generate consensus on ethical guidance for CGT HIV cure research. Despite these limitations, our study generated preliminary considerations to guide the blossoming research of CGT towards an HIV cure.

## Conclusion

Our qualitative formative study identified preliminary ethical and practical considerations towards the goal of achieving a cure for HIV through CGT. Rapidly evolving CGT towards an HIV cure is accompanied by a host of ethical and practical challenges. To minimize risks to potential participants and facilitate the translation of scientific advancements from the bench to the clinic, CGT HIV cure research must be thoughtfully developed and implemented. More research will need to be implemented with more diverse respondents in various settings, including resource-limited locales. To protect the public trust in CGT HIV cure research, ethical and practical considerations should be periodically revisited and updated as the science continues to evolve. Increased public engagement around the scientific potential of CGT will also be necessary.

## Supplementary Information

**Additional file 1. Supplementary Table 1**. Preliminary Ethical and Practical Considerations for Cell and Gene Therapy Towards an HIV-1 Cure – Selected Quotes (United States, 2020 – 2021).

## Data Availability

All relevant quotes have been included in the results section and in Additional file 1: Table S1.

## Abbreviations

AAV: Adeno-associated virus
ART: Antiretroviral treatment
ASBMT: American Society for Blood and Marrow Transplantation
ATI: Analytical treatment interruptions
CAPS: Center for AIDS Prevention Studies
CAB: Community advisory board
CAR: Chimeric antigen receptor
CCR5: C–C chemokine receptor type 5
CGT: Cell and gene therapy
CIRM: California Institute for Regenerative Medicine
CMC: Chemistry, manufacturing and controls
CRISPR/Cas9: Clustered regularly interspaced short palindromic repeats/CRISPR-associated
CRS: Cytokine release syndrome
DARE: Delaney AIDS Research Enterprise
DNA: Deoxyribonucleic acid
DSB: Double stranded break
DSMB: Data safety monitoring board
FDA: Food and Drug Administration
FIH: First-in-human
GGTI: Global Gene Therapy Initiative
HARP-PS: HIV + aging research project-palm springs
HIPAA: Health Insurance Portability and Accountability Act
HIV: Human immunodeficiency virus
IBC: Institutional Biosafety Committee
ICANS: Immune effector cell-associated neurotoxicity syndrome
IND: Investigational new drug
IRB: Institutional Review Board
PLWH: People living with HIV
SCID: Severe combined immunodeficiency
TALEN: Transcription activator-like effector nuclease
UCSF: University of California San Francisco
UNC-CH: University of North Carolina at Chapel Hill
ZFN: Zinc finger nuclease
