# Supplementary material for: Ethical and practical considerations for cell and gene therapy toward an HIV cure: findings from a qualitative in-depth interview study in the United States
Source: BMC Med Ethics. 2022 Apr 9;23:39. doi: 10.1186/s12910-022-00780-1 (PMC8994300; doi:10.1186/s12910-022-00780-1)
Supplement: Supplementary file 1 — Additional file 1. Supplementary Table 1. Preliminary Ethical and Practical Considerations for Cell and Gene Therapy Towards an HIV-1 Cure – Selected Quotes (United States, 2020 – 2021). [file 12910_2022_780_MOESM1_ESM.docx]

Supplementary Table 1: Preliminary Ethical and Practical Considerations for Cell and Gene Therapy Towards an HIV-1 Cure – Selected Quotes (United States, 2020 – 2021)

| **Themes and Sub-Themes** | **Participant Number** | **Informant Type** | **Additional Quotes** |
| --- | --- | --- | --- |
| 1. **Perceptions of CGT and Benefit/Risk Considerations** | | | |
| **1.1 Perceived Benefits of CGT Towards an HIV Cure** | | | |
| Possibility of single-shot regimens | 05 | Community member | *I also just think that [CGT is] a better way, you know, more complete and better way to really get into the body… to change what's wrong and fix HIV… From the get go, it's going to go in there, the gene therapy, and change what's broken.* |
|  | 05 | Community member | *With the trial that I was in, there was only one infusion, so I had one treatment, basically for life.* |
|  | 08 | Community member | *It would be great if we could give someone a treatment once, like the ideal of a shot in the arm, and bam, we're taking care of HIV.* |
|  | 03 | Biomedical researcher | *If there were to be a single shot in the arm that was more accessible, more palatable, more likely carried out in such individuals, that would be a boon to them and to their partners or whomever they might spread virus to.* |
|  | 11 | Biomedical researcher | *To be able to come up with a one-shot treatment, for example, the AAV express-bNAbs combination where you deliver it, it's expressed in the patient cells, and then they're basically done, for some, maybe forever or however long that expression continues. So, I think that's the main benefit is the longevity.* |
| High potential to lead to complete elimination of HIV | 02 | Biomedical researcher | *[I]f you could replace the immune system with cells that are not able to be infected, you would be cured, you wouldn't need drugs. With the latency-reversing things, you're actually turning the virus on, so there's risk associated with that. You're also stimulating cells and there's risk associated with that. So, if you can treat with a cell-based approach that is not harmful and is permanent, you could get around those issues.* |
|  | 09 | Biomedical researcher | *The things we've tried in the past haven't really worked terribly well. [W]e need new technologies [and] new abilities to alter the immune system or alter how HIV is able to hide in the body for us to get a cure.* |
| Scientific advancements for other molecular diseases | 06 | Biomedical researcher | *There's a lot of other genetic diseases and some cancers where the targets of the therapy are very difficult to find, and I think HIV… could provide a lot of benefit for the ways that we target [treat] other people with other viruses and other molecular genetic diseases.* |
| **1.2 Perceived Risks of CGT Towards an HIV Cure** | | | |
| Clinical risks of CGT  (i.e., side effects) | 05 | Community member | *I think that the risks that everybody talks about is cancer or malignancies being, you know, caused by changing a piece of the DNA.* |
|  | 08 | Community member | *You have those off-target edits, which can be causing cancer, and that might not happen for 20 years.* |
|  | 16 | Community member | *You may not get a response that you're looking for, you may just get real sick… maybe you get too suppressed or too toxic or something.* |
|  | 02 | Biomedical researcher | *If you put gene-modified cells into the body, there is a potential to have one or two… of those cells become malignant, and that would be hazardous.* |
|  | 09 | Biomedical researcher | *Anytime you modify the genome, there is a possibility of something untoward going after.* |
|  | 17 | Biomedical researcher | *For CAR T-cell therapy, people tend to worry about cytokine release syndrome.* |
|  | 19 | Biomedical researcher | *There's something called insertional oncogenesis, that just the idea of a viral vector integrating itself into the host DNA could induce a malignancy, by knocking out a tumor suppressor gene or something.* |
| Risks of having to replace immune cells | 02 | Biomedical researcher | *The other risk is how much of the immune system you would have to replace in order to affect a cure. So, if you have to replace all of it, that means you have to eliminate all that first and then rebuild it, and so there's a risk there as well.* |
| Unknown risks of CGT | 05 | Community member | *They are questionable, we don't know about some of them, we're still learning… And it's going to take people that are pretty brave… to enroll in trials like this to kind of get over the things that we don't expect or we don't know.* |
|  | 09 | Biomedical researcher | *But, first in human means it's first in human and, so, we don't know. That's why we're doing the trial. And so, I think that's an inherent risk that I think people who participate in the trials really have to understand. That, look, we've thought about this. We did everything we could with it, but we don't know. That's why we're doing the trial. If we knew the answer, we wouldn't be doing the trial.* |
| Risk of transmitting HIV to sexual partners during ATIs | 09 | Biomedical researcher | *The real risk is someone becoming viremic without knowing and then could potentially transmit the disease to somebody else… To me, that is the most tangible risk in this type of therapy... But… the only way we know if it works, is by taking people off their medicine… To me that's the scariest adverse effect… is that somebody unwittingly transmits the disease to somebody else because of the trial they're on.* |
| Risks of interventions not having intended effect | 17 | Biomedical researcher | *The risk is that you may develop this therapy, and it may not work as you intended in the participant.* |
| Creating false hopes and expectations | 16 | Community member | *Potential risk is that your hopes could be easily shattered.* |
| Financial costs of CGT | 06 | Biomedical researcher | *The financial costs of cell and gene therapy is something that, it's a great focus for us… And from a scientist's perspective, sometimes we feel a little helpless in that regard because we're just trying to make these things work, and we're not really the economists.* |
| Burdens on study participants | 15 | Biomedical researcher | *Look you have to take so much time off of your life to come here.* |
| **1.3 Ensuring Acceptable Benefit/Risk Balance** | | | |
| Minimize risks as much as possible | 14 | Bioethicist | *What are we going to learn from doing the study? Have we made the risks as low as we can make them? At a certain point, if you've designed the best science you can design, and you're going to learn something really, really valuable, or you have the potential to learn something really, really valuable from it, and you've really thought carefully about the way you're doing it, and ways that minimize the risk…* |
| Implement research in incremental steps | 05 | Community member | *You want to make sure you're doing it slowly, within reason… You study a product in small groups and then you… go to larger groups…* |
|  | 11 | Community member | *I think you move carefully, basically. I think you move incrementally, stepwise, and try to minimize your risks, so that you're not adding combinations all at one time, which might cause a bad adverse reaction. So, I think taking a careful stepwise approach is the best approach, safest approach… I think that going, again stepwise, so you're only adding one new agent at a time and doing incremental dose escalations is the best proven method for avoiding bad effects.* |
| Ensure adequate regulatory review | 07 | Biomedical researcher | *We have these regulatory bodies. One of the things we have to have in any genes or cell therapy trial or research project is what's called an Institutional Biosafety Committee. So, we have IRBs for human subjects research, anytime gene or cell therapy is involved, we also have to have an IBC.* |
|  | 17 | Biomedical researcher | *Giving that to the FDA, some kind of regulatory framework to scrutinize your work and to also help you if you are having some gaps there in terms of the benefit to risk…* |
| Implement robust risk mitigation strategies | 17 | Biomedical researcher | *Make sure that you put in an appropriate and stringent risk mitigation strategy for the trial.* |
| Maximize scientific data around safety and efficacy | 06 | Biomedical researcher | *I think having as exhaustive data as possible, looking at the safety of the approaches, as well as how well it's likely to work… But I think if the so-called efficacy, or how well it's working in the cure, if that's very high, and we think that it's feasible to administer these therapies in one way or another and the toxicity is low, I think it just comes down to what the benefit is for an otherwise healthy person living with HIV.* |
|  | 17 | Biomedical researcher | *You need to make sure that, when you start thinking about these therapies, you have the right preclinical studies to support the efficacy and safety of your therapy.* |
|  | 19 | Biomedical researcher | *The first thing we really need to do, I think, is to be extremely fastidious with all of the preclinical experiments.* |
| Ensure constant vigilance around acceptable benefit/risk balance | 08 | Community member | *But I think we just need to always be ever-vigilant, for the sake of our fellow human beings, our brothers and sisters, our wives, our husbands, our lovers, who have HIV, and who are gonna be living desperate lives in some sense, because of the way HIV is still stigmatized. They're gonna be willing to do things that you wouldn't want them to do. And so, therefore, they are vulnerable to research, and to… unethical research.* |
| Be clear about expectations | 16 | Community member | *The way to manage it is to be honest about what expectations you do know about.* |
| Consider potential benefits to humanity if proven effective  (social value) | 09 | Biomedical researcher | *For HIV disease, this is truly altruistic. The people who participate in our trials, they're not doing it because they think they're going to get cured. I think in the long run they're doing it because they hope there is a cure… I think from a risk-benefit point of view, it doesn't make sense. But from a humanity point of view, somebody who's angry at their disease and how it's… stigmatized them, caused them to take all these medicines, I do think they do want to fight back a little bit and say, "Okay, I want to work towards a cure."* |
| **1.4 CGT Strategies Perceived to be Unacceptable for Human Testing** | | | |
| Germline editing | 11 | Biomedical researcher | *I think that genetic manipulation of germline cells, like they did in China, is out of bounds and too risky.* |
|  | 17 | Biomedical researcher | *I think when people start to think about editing, like gene editing strategies, perhaps using CRISPR or other types of similar approaches at the germline. That's when I think things become a little bit dicey.* |
| Allogeneic stem cell transplants in otherwise healthy volunteers | 06 | Biomedical researcher | *At this time, …with our current technologies, I would say stem cell transplantation is too risky because… we would need to be so aggressive with that therapy [that] the risk benefit analysis or ratio wouldn't be in balance.* |
|  | 11 | Biomedical researcher | *I think that stem cell modification in otherwise healthy patients, or participants living with HIV or in this indication, I think healthy volunteers. I think at this time, it's of the highest risk, anything involving stem cells. Now, if you have cancer and HIV, then it becomes a more acceptable, different calculation.* |
| Intervention that could lead to debilitation or death | 05 | Community member | *I don't want to have some kind of side effect that makes me debilitated in some way… a side effect that is enough to make me so sick that I don't want to just function.* |
| Unacceptability risks depend on individual volunteers | 07 | Biomedical researcher | *I mean, it's a tricky question because, again, I think each individual person has to evaluate what risk they're willing to accept in exchange for a benefit.* |
|  | 08 | Community member | *It would depend on the person that I was talking to. Like, in other words, if someone wanted to do a CAR T-cell therapy for HIV I would not tell them not to do it, but I would wanna know more about their own situation, their health and things.* |
| Science should not be restrained if conducted within ethical boundaries | 12 | Biomedical researcher | *I think that, while we keep our ethical considerations and approval, everything is game. I don't think we should limit science. We should protect subjects [participants], but we should not limit science. And a lot of people, under the disguise of protecting patients, they're really biased. And sometimes it's just as simple as not accepting a new technology.* |
| **1.5 Additional Ethical Considerations for CGT Approaches Towards an HIV Cure** | | | |
| Safety maximization | 04 | Biomedical researcher | *So if we can't represent this, then really the driver of ethics through the rest of it is our personal commitment to extreme self-criticism and intense safety testing because nobody else is going to be able to raise the concerns about product safety that we can raise ourselves.* |
|  | 17 | Biomedical researcher | *Well, of course, when you think about the ethical point, you want it to be safe. Definitely, you don't want it to create any harm to participants when you put them in there.* |
| Fair participant selection | 04 | Biomedical researcher | *First of all, you're obligated to recruit volunteers during the process development that reflect the population you intend to use this in. During our process development work, we recruited HIV positive individuals from diverse racial backgrounds and paid attention to the male-female balance and made sure that those people, to the extent possible, would actually qualify for the future phase one study.* |
|  | 12 | Biomedical researchers | *Then, I think the other thing is, typical things about pregnancy, not to do it in pregnant women because of the chance of this being incorporated into the baby. And then the typical precautions for the at-risk population. So, the pediatric population, you have to have special considerations there, and then people who cannot make their own decisions, so mental health patients, people incarcerated, all these things...* |
| Distributive justice | 08 | Community member | *So, you have always… in the background, a question about is it ethical about going into cure when people don't even have access to treatment and 15 million people are facing death from HIV? Shouldn't we be giving more money to get that treatment everywhere?* |
|  | 17 | Biomedical researcher | *We also think about the cost and, if we have to make this universal therapy, how can we apply it to maybe resource-limited settings as well?* |
| Robust informed consent process | 08 | Community member | *And that just means it's essential for us not to inflate our promises of personal benefit, and to ensure that anyone who's doing research and the perspective [prospective] participants in their studies, really appreciates this uncomfortable reality… [that] we have imprecise consent forms that say things that maybe they shouldn't be said that way. If we fix the consent forms, the whole consent process is so legal and so beyond human interactions, that people just shut down and will sign. Really, people do not look at the consent as a way of consenting someone. It's like a legal document, and it needs to be changed. And the fact that people are so resistant to changing it, and letting it become something that you can do via video, shows you that they really are not about consent. It's a legal document.* |
|  | 16 | Community member | *There's ethical considerations into how you talk to somebody about treatment and the risk and benefits.* |
|  | 06 | Biomedical researcher | *The number one thing is to give a prospective patient or clinical trial participant all of the resources they need to easily, and I think that's an important point, easily understand what's going on... And I don't think that, personally, there's anything wrong with just being very frank about the state of the technology right now.* |
|  | 09 | Biomedical researcher | *I'm more to getting people in the trial who are knowledgeable, that really have done their homework, that really understand what they're getting into… I'm more interested in the type of person who would really want to do this trial, and they're doing it for the right reason, and they spend the time to really understand what is going on in the trial.* |
|  | 10 | Biomedical researcher | *So, a lot of these concepts… are captured in an informed consent document. But a document is just a document. We live in a world where we all scroll through the terms and conditions and sign off at the bottom. And I would be very anxious to know that informed consents are not just a document, that it's something that colors how patients are recruited and how they are educated about the process, so that we never have somebody sign up for a trial who just doesn't really understand the potential risk they will be facing, as well as the potential benefit.* |
|  | 11 | Biomedical researcher | *In terms of ethics, I think you just need to make sure that the informed consent makes it clear that the participant understands what risks they're taking… I think that information is the most important thing to convey. But I think, if you have altruistic participants in a study, I think it can be done as ethically as any other study.* |
|  | 15 | Biomedical researcher | *But because the consent is complicated, the therapy is complicated, the consent is complicated and it's difficult for the sites to explain all this to the participants.* |
|  | 19 | Biomedical researcher | *I think there's also a perception there, like I think it's something where individuals that are considering this therapy are really going to have to understand that they have some active gene therapy modality that's being delivered inside them. They need to be fully informed about that and what that means, that something is actually going in and essentially modifying their DNA inside them. That's a big deal, I think.* |
| Ethics of embryonic stem cells | 02 | Biomedical researcher | *So, one is the origin of the cell. Does the cell come from the same individual, which has less of an ethical ramification than if it comes from somebody else? Or people now are working on embryonic stem cells and induced pluripotent stem cells. An embryonic stem cell comes from an embryo; that is somebody else or would have been somebody else and there's some ethical issues with that. Induced pluripotent stem cells have less of that issue because that is taken from the same individual and then converted into a stem cell and then re-manipulated. So, that has less of an ethical issue. But using somebody else's cells could be some of the ethical things that we're thinking about.* |
|  | 04 | Biomedical researcher | *I am not opposed to embryonic stem cell research personally but, as a responsible person running a company, I avoid that area. So, that's an ethical consideration that sort of sits out there in general.* |
| Ethics of HIV analytical treatment interruptions (ATIs) | 08 | Community member | *I think, through all cure paradigms, is the treatment interruption question. Because to know if… treatments are working, at least currently, we're gonna need to do some sort of treatment interruption, and exposing [participants] to potential risks with that procedure, making them sick, perhaps, again, not immediately, but in the future, because of a viral rebound.* |
|  | 19 | Biomedical researcher | *Because at this point, I think the only reliable thing that we can do to see if there's been any meaningful benefit is just to stop antiretroviral therapy and see if there's any impact on how long it takes for the virus to come back. But as you know, that's not very satisfying. Number one, it's not very scalable because it's not without harm. There're studies now suggesting that treatment interruption does not have any lasting, negative consequences to an individual in terms of disease progression or lasting immunological consequences. But there's some undeniable risk, in terms of an individual might be infectious for some period of time before they're aware that their virus has rebounded… So, it is not without harm to do these analytical treatment interruption studies. So, I think one of the key things for the cell and gene therapy cure field, and then just the HIV cure field in general, is we need to come up with biomarkers that we can measure conveniently.* |
| Ethics of CGT development | 01 | Community member | *Companies may decide that they don't want to continue to benefit [or]… that they don't want to continue to work in HIV because … it is hard… I can see why your business model might suggest you move on to another disease, but we need to think about some method [whereby] the whole community receives some later benefit from having helped these companies actually achieve the safety signal in their product that they needed to be able to move on to later work.* |
| **1.6 Considerations for First-in-Human (FIH) CGT HIV Cure Trials** | | | |
| Robust pre-clinical safety data | 02 | Biomedical researcher | *Again, you need adequate testing initially; so, that would certainly require animal modeling at some level because in vitro studies really don't get at the safety profile… But I would have to be pretty certain that there is a direct benefit [efficacy] with animal models before I would go to clinical trial.* |
|  | 06 | Biomedical researcher | *There's going to be a lot of requests for safety data and having a very clear idea of what the toxicity risks are that are involved.* |
|  | 17 | Biomedical researcher | *So, to me, that's the major ethical consideration is the safety of this product, to make sure it's safe. And then, anything we do in the downstream of infusing the product. How can we also make sure participants are safe and they're being evaluated and being taken care of?* |
| Reliance on regulatory authorities (e.g., U.S. FDA’s IND process) | 02 | Biomedical researcher | *You really have to have FDA oversight and data safety monitoring boards watching closely.* |
|  | 11 | Biomedical researcher | *I think the FDA will make sure that you have, for example, off-target genetic analysis built-in to the program and pre-clinical data on off-target effects. I think that'll be a part of the safety package. So, I don't have any specific ethical concerns as long as that'll be part of what's monitored.* |
|  | 12 | Biomedical researcher | *So, going back to your question, how much for clinical data you need, I think as much as the regulators want, correct? Because that's the only way you're going to get it pass.* |
|  | 17 | Biomedical researcher | *By working with regulatory frameworks like the FDA, they are going to be the ones to come back to you and say, “Hey, you need to follow this guidance that we put out here for you to be able to say, check, you did a good job on evaluating the safety and efficacy.” And if not, they're going to kind of redirect you back to the drawing board to get it done. So, I think if we have those two, you may think you've done enough, but you need to have someone that can scrutinize that work that is an expert in the field and lets you know, yes, you've done enough and now you can proceed in humans.* |
| Early involvement of PLWH and compensation for research-related injuries | 08 | Community member | *You should have the people of that population involved from the beginning…; patients really need to be involved at the trial level. If it's first-in-human you need to make sure that you have at least one person who lives with HIV involved at the get-go…, not a tad later on…, because I think they can help you with the acceptable balance of risks and benefits… Compensation needs to be thought about… if something goes wrong [the participants] should definitely be compensated.* |
| Limitations of current animal models | 02 | Biomedical researcher | *Well, there's a lot of testing in nonhuman primates, and there's pluses and minuses for that. That's the closest model we have to people, but the virus is not HIV, so it's a little different than HIV. You can engineer SIV to have bits of HIV in it which may be a closer model, but is still not perfect, but that's really the closest thing we have to modeling prior to going to people. There's humanized mouse modeling which is what our lab does which uses the right virus, but it's not as robust as a nonhuman primate.* |
|  | 03 | Biomedical researcher | *There is no real animal model that can be used to predict safety or efficacy, I don't believe, in humans. I know that people say that there are, but it's really been proven more likely wrong than right over the decades.* |
|  | 09 | Biomedical researcher | *That's a great question because, so far, the animal models have, at least in my opinion, have largely failed us, right? We have cured HIV in monkeys lots of times but that success has not translated to humans. And there's lots of reasons for that: one, it's a different disease and, two, as humans and researchers, we like things to work, so we design our experiments so they have a better chance of working… In humans, we don't have that luxury.* |
|  | 10 | Biomedical researcher | *We see preclinical studies, including animal models, [and] we always recognize the shortcomings of these models… So, I think the challenges of extrapolating from preclinical studies, including animal studies, to the reality and HIV positive individuals is understood, and people don't over promise.* |
|  | 11 | Biomedical researcher | *It doesn't necessarily predict, in terms of efficacy, what's going to happen in the human*. |
|  | 19 | Biomedical researcher | *I actually think one of the key challenges for cell and gene therapy within the context of HIV infection, and actually, for the HIV cure field in general, is that there aren't really that many great choices for preclinical animal models... There's hardly anything where the model is satisfactory, in that it really recapitulates a lot of the key features of the host virus interaction in the real McCoy, a human being… I think a real key step, in terms of making sure that when we go into an infected individual [PLWH] with one of these therapies, that we're doing something where the benefit to cost ratio is satisfactory, is to really do everything we can to be beef up and develop these preclinical models, to make sure that we can rigorously evaluate them before we take them into the clinic.* |
|  | 19 | Biomedical researcher | *So, we think that probably the closest model would be the nonhuman primate models but the problems you have there, number one, aside from any ethical considerations, [is that] they're very, very expensive experiments, and so usually you can only end up testing something in like five or six animals, which is really a woefully small sample size. And so it's really hard to draw any generalizable or statistically rigorous conclusion from any of those nonhuman primate models. And then, of course, the idea of doing these experiments in nonhuman primates… brings up other ethical considerations.* |
| Robust review of pre-clinical data | 08 | Community member | *We should insist before anything goes into humans that it is published, that animal data, or that pre-clinical data has been published, so that others can make independent judgments about the quality of the work, before it goes into humans… They [the investigators] can't just keep that data private.* |
| Well-designed and supervised FIH trials | 08 | Community member | *Safety measures have to be built into the trials, and we should have lots of people looking at those. You know, like, there are different things that they can do about the way they administer things in first-of-human trials. Like, maybe they administer it in a slower way, or maybe you just give it to one person, and then you wait to see… the, you observe the person before you put it into a second person.* |
|  | 09 | Biomedical researcher | *I'm a big fan of small, well-supervised clinical trials to figure out what's going on.* |
|  | 10 | Biomedical researcher | *But, also, we understand that much of the progress we're going to be making will have to come from incremental steps and knowledge that we get from doing clinical trials in people… I think a really important thing is to make sure that the design of the clinical trials it's done as well as we possibly can, so that any information we can get from that trial can be harvested…* |
| Need for better guidelines for FIH trials | 08 | Community member | *I think there needs to be better guidelines for first-in-humans, especially if it's involving some sort of high-risk product, and I think it needs to be broken down and made transparent how these guidelines are supposed to work... like the mode of action that the technology's gonna use, the nature of the target that it's focused on, and how the animal models, then, are relevant to it. I think those guidelines need to answer those things before we put it into humans.* |
| 1. **Safeguards and Risk Mitigation Strategies** | | | |
| **2.1 General Safeguards for Developing CGT Approaches Towards an HIV Cure** | | | |
| Clinical trial design issues | 04 | Biomedical researcher | Thoughtful inclusion/exclusion criteria: *We have pretty narrow inclusion-exclusion criteria to try to have people really as healthy as possible that enter the trial at first.* |
|  | 15 | Biomedical researcher | Thoughtful inclusion/exclusion criteria (to match participant population): *Look at specific inclusion criteria and try to find what are the best candidates for a specific therapy.* |
|  | 04 | Biomedical researcher | Staggering participants: *Staggering interval in the trial. The staggering interval means… the time between infusing person A and infusing person B.* |
|  | 10 | Biomedical researcher | Lower initial doses: *The early trials will have to go in with low doses and almost zero expectation that this could be successful. Because what we're looking for is, what are the consequences of delivering anti-HIV gene editing tools for example, into somebody?* |
| Manufacturing and transportation safeguards | 09 | Biomedical researcher | *There's obviously lots of safeguards in terms of the actual products that we use: basic biology that they're sterile, that there's this identity testing. But those are pretty straightforward to be honest with you and, I think they're sufficient for what we're doing. If the product passes the criteria that we set out for it, I feel good about putting it into the people. I don't think we need any more stuff for that.* |
|  | 19 | Biomedical researcher | *So, even if that's an ex vivo cell therapy procedure, there needs to be quality control, so that when you do the gene editing ex vivo, before you infuse the cells back in, we need to be able to enrich or purify for the cells that we've edited, and make sure that we discard any cells that received edits at the wrong place.* |
| Close clinical monitoring (including long-term monitoring) | 07 | Biomedical researcher | *In team research, you have to have that person [who] is willing to say, “Okay, what's the worst possible outcome? Are we doing everything to mitigate the risk for that? And are we willing to accept the outcome if that risk comes to fruition?”* |
|  | 11 | Biomedical researcher | *Well for gene therapy… monitoring off-target effects, genetically sequencing and looking for off-target effects is critical. In terms of cell therapies, you're going to need to monitor the immune response and make sure that you don't get a cytokine storm type response. I think those are the main concerns. You could also have an anti-drug or anti-antibody type response, which will limit the efficacy, but it's not so much a safety concern; it's more of an efficacy concern.* |
|  | 17 | Biomedical researcher | *But then when we start talking about CRISPR, we need to think about, inside the cell, what is really happening? Are there being any genetic changes to the cell that affect neighboring genes, genes that reside close to where the integration occurs? And if it does, what does it mean? What's the implication? Would this lead to some type of clonal outgrowth which could lead to cancer?* |
| Adequate training of research staff | 04 | Biomedical researcher | *We conduct onsite training. We make sure that we have medical experts available to these sites who are professionally dealing with those complications already and understand them. And we make sure that the sites have ready access to the medications needed to reverse these syndromes*. |
| Cumulative body of evidence (safety safeguard) | 15 | Biomedical researcher | *The more experience you accumulate with cell and gene therapy the more you're able to identify the safeguards. You are building that body of evidence that helps you make the therapies safer and safer.* |
| Monitoring for potential conflicts of interests | 07 | Biomedical researcher | *I have to have a doctor who has zero interest in whether or not this succeeds scientifically who is the person who's running that trial. So, they have to have no conflict in doing this evaluation, and they have to be sincere in their need to care for the patient in terms of how that would look.* |
| Community involvement | 08 | Community member | *I think what safeguards need to be in place… when it comes to cell and gene therapy, community needs to be involved. I think the HIV part we have community involved, but I'm not sure that all cell and gene therapy for HIV is going to come through the HIV arena… Everyone should have community involvement, and seek the communities that they're wanting to help, and having them be a part of the process, welcoming them in, even if you're not sure how they can add to your basic science.* |
| **2.2 Safeguards for Combining CGT Approaches** | | | |
| A cure for HIV will require a combination of approaches | 14 | Bioethicist | *I would actually have thought that a lot of… candidate interventions might actually be combined cell and gene therapies. Whether it is autologous cells that are taken out, medically manipulated, [i.e.] modification of the CCR5 receptor [or] whatever it might be, and then reinfused back…, that's a combination of cell and gene therapy, or whether it is somehow… off the shelf stem cells that are genetically modified, I would imagine that a lot of potential interventions might be in effect combined interventions.* |
|  | 08 | Community member | *It's gonna take a combination of something. I don't think just any one thing... It'll be a process.* |
|  | 16 | Community member | *But I think communicating again that, sure, I mean for me, it's highly likely that it's more than one intervention that will cure HIV.* |
|  | 15 | Biomedical researcher | *So, it's always going to be cell and gene therapy combined.* |
| Safety of combination CGT products | 05 | Community member | *You just might want to make sure that they're both safe, and… you obviously want to determine if there's a synergistic effect which can be lethal, can be dangerous, but also could be effective*. |
| Continued investment in pre-clinical work | 09 | Biomedical researcher | *I think if there was anything that was terribly dilatory, you would see it in the non-human primates model.* |
| Informing participants of risks of combinatorial CGT approaches | 16 | Community member | *I mean the safeguards are just that people need to know that about the risk that they're taking as much as what is now. People need to be able to articulate that. Because you got to remember the one thing that often draws people to research is hope. Not everyone approaches it from the same way, but a lot of times people are like really latching onto hope that this is going to be something that is going to cure me or like be a home run of some kind.* |
| Transparency around potential risks of combinatorial CGT approaches | 08 | Community member | *But again, if it came to a clinical trial, that's when I would wanna have transparency. So, I guess that's the other thing I would say as a safeguard. And when you're getting ready, and you're designing that, not only do you have community involvement, but once you have that finished protocol, that you are introducing it to your constituents, to your participants, to the populations, so they can look through it and they can ask a million and one questions before you start enrolling. I think would be the key.* |
| Combination CGT products may depend on health status of patients/participants | 12 | Biomedical researcher | *I think it depends on the status of the patient and the risk-benefit ratio… it’s all about risk-benefit. Should I try, let's think about three different type[s] of gene therapies together... on a patient that is stable enough for [anti]retrovirals for years? Probably not... But would I treat a patient that is sick all the time? Yeah.* |
| Robust community involvement | 15 | Biomedical researcher | *So, that's why after so many years in this field, I really feel like it should be, and I admire some for doing that, there should be no phase one first in human [trials] without input from the community.* |
| **2.3 Mitigating Off-Target Effects of CGT Interventions** | | | |
| Improved targeting during engineering process | 02 | Biomedical researcher | *Well, one way to prevent off-target effects is to have better targeting of the approach. So, as the technologies develop, these things become safer. So, when we talked about lentiviral vectoring, when you throw a lentivirus gene vector system onto cells, that virus integrates randomly so it can hit, essentially, randomly. It can hit all kinds of different spots in the genome, and those could cause problems depending on where it inserts. If you have an approach that targets a knockout, which is specific for one gene, in theory, that should be less risky because you're just hitting that one gene. But now, you have to do enough tests to show you don't have an off-target effect in the genome.* |
|  | 04 | Biomedical researcher | *We work extremely hard to make sure that we are as on-target as possible. That's in the process engineering. If you look at our final product, about 80 – 85% of the vector sequences are only in the target[ed] cells. So, the spill over into other cells is absolutely minimal.* |
|  | 09 | Biomedical researcher | *At least for CAR therapy for HIV…, the target's pretty well defined. We're going after [the] HIV envelope; we're going after something that's solely HIV… I'm pretty sure we could design therapies that are on-target.* |
|  | 17 | Biomedical researcher | *It is because you have this therapy that you're putting in the body, at least for CAR T-cell therapy, and the whole idea is that it will seek out the affected cell or the pathogenic cell and destroy [it]. And so, in order to do that, it has to be specific. So, definitely during those pre-clinical studies, and the FDA will request this, is that you have to do some type of specificity testing to assure that your product doesn't have any off-target effects in the body.* |
|  | 19 | Biomedical researcher | *The beauty of this whole CRISPR revolution thing, to put it succinctly, is that CRISPR-Cas9 enzyme or family of enzymes, are way, way, way, way, way, way more programmable than their predecessors that we used in the gene therapy field… That being said, even though CRISPR-Cas9 is so programmable, mistakes happen, and they still happen.* |
| Extensive testing for off-target effects | 02 | Biomedical researcher | *Those techniques do exist where you can monitor whether you've cut somewhere else in the genome and at what frequency. And, typically, you do get some off-targeting of an approach like that, and you'd have to figure out what the risk is with the specific type of off-targeting you get and the specific frequency of off-targeting you get of that approach.* |
|  | 07 | Biomedical researcher | *So the first thing would be what happens if the editing occurs elsewhere in the DNA that's not CCR5 and what potential impact does that have in the same cell? … But you could also think about what happens if we get editing at CCR5 or someplace else in the DNA, in a cell that's not the cell that we're trying to do it in, and what effect does that have? … If you're coming up with a new gene editing strategy for HIV, you're going to be asked to document in every experimental setting that you evaluated off-target and you tried to understand as best you could what the risks or side effects or adverse events were that were associated with that and how you're defining it… You're going to have to have a strategy in place that's going to say, “How are you going to monitor the first people who agree to be part of this study? How are you one going to inform them of the potential risks associated with this?” And two, “How are you going to monitor for those risks and new risks that we haven't anticipated?” … And then there's a reporting requirement for how quickly we have to transmit information regarding when new risks or adverse events are identified… And then we'd have to finalize our report based on that and give what we call an attribution, meaning do we think that this was related or not.* |
|  | 09 | Biomedical researcher | *Now, is it possible that some weird antibodies could cross-react? It's possible, and so you can do some what's called tissue sampling. There's these banks of tissues, human tissues.* |
|  | 11 | Biomedical researcher | *I think it's part of the pre-clinical package to have done studies and statistics, to look at the frequency of off-target effects and what genes are affected by those, and that will help to inform. At some point, it becomes a mathematical risk assessment to help predict the likelihood that you're going to have some off-target effect.* |
|  | 15 | Biomedical researcher | *So, off-target is being mitigated by looking very deeply into the genome and figuring out if the modifications popped up somewhere else. And the techniques are getting better and better.* |
|  | 19 | Biomedical researcher | *Having ways to safeguard ourselves against the off-target effects, so there's got to be some clear diagnostics in place to make sure that we know when… editing has occurred where we don't want it.* |
| Monitoring for off-target effects | 03 | Biomedical researcher | *With in-vivo [inside the body] therapy, you don't have the opportunity to selectively hit one population of cells because all cells are equal partners for potentially being hit. So there you have to look for gene modification of the type that you've introduced in cells that are off-target. You would look for it by not just drawing blood, but also by taking careful biopsies of the places in the body where those genes might go.* |
| Challenged scientific concept of off-target effects | 12 | Biomedical researcher | *But the truth is that we are looking only one pathway when we do those things. Everybody responds to any therapy in different ways. So, these targeted therapies are never targeted. We see it in oncology. That's why this comment of off-target effects talk because you have all this inhibitors of this gene, inhibitors of this other gene, and things are precisely made against that DNA and that you'll get 20% off-target effect. Sometimes off-target effects are even better… I'm not going to really worry about the cell and gene therapy, because they are much better targeted than the drug. With the drugs, we have them interpreted as one drug attacks one pathway, and usually doesn't. That's a reason why cancer cells are so good growing again: …you block this pathway? I'll find another pathway. You block that one? I'll find another one.* |
|  | 19 | Biomedical researcher | *So, whatever we can do to reduce the off-target effects and then increase the specificity of the approach, I think is critical… Let's say that wasn't even an issue, so all we were getting was on-target editing, there's still risks there. So, I'll bring it back to this idea of using gene therapy to get rid of the CCR5 entry receptors. So, the good news is that, if we could effectively get rid of the CCR5 entry receptor, you would largely, not completely, but you could largely protect cells from getting infected by HIV. But, of course, nature didn't put the CCR5 gene and the CCR5 protein there to help the virus get in; it's got a function within the host… But one of the limitations is that the people who have that gene deletion, they actually developed in utero with that gene deletion, so there was probably a whole developmental and immunologic remodeling and remapping that happened during the very early stages of life, during the earliest stages of life, that compensated for that gene deletion. So, that might be a very different situation than if you take a fully mature adult individual and then get rid of that gene. There might be some immunologic consequences.* |
| **2.4 Mitigating Risks Associated with Long-Term Duration of CGT Interventions and Risks of Immune Overreactions** | | | |
| Desired duration depends on the specific investigational product and mechanism of action | 03 | Biomedical researcher | *How do you make sure that the duration of therapy is long enough to be good but not so long as to be bad? ... Our targets in-vivo are selectively designed to be long-lived, asymmetrically dividing, so-called stem cells. And the reason for that is because their biology is well known and we want the effect to be a single shot that lasts for a long time.* |
|  | 04 | Biomedical researcher | *Other people who are, let's say introducing things like CRISPRs, they have talked about ways to control duration of this, but I don't know that there's anything really solved.* |
|  | 06 | Biomedical researcher | *If we had an antibody-based approach where we had some vector that just constantly pumped out antibody for the life of a patient, I'm not sure that that... I don't think there's any data right now that suggests that would need to be turned off.* |
|  | 10 | Biomedical researcher | *There the therapy is based on long term production, so, you want to choose a gene therapy vector that's safe and innocuous, and AAV vectors, so far, look to be innocuous, but they can survive and be producing that antibody. So, it's going to be important to choose the right type of therapy and, therefore, get the appropriate duration, whether it's hours or years.* |
|  | 12 | Biomedical researcher | *I think it depends what it is about.* |
|  | 17 | Biomedical researcher | *I think for the therapies that we're developing, you want them to last in the body but... you want them to be at a low level when the disease is under control, and just kind of hang out there so when there is some resurgence of the virus then it just kind of quickly balances back.* |
| Ways to control the duration (e.g., safety switch) | 02 | Biomedical researcher | *There are genetic ways to eliminate manipulated cells. So, there are genes you can throw in that make a cell that has that gene susceptible to a toxin or another drug that you would normally be susceptible to. So, if you're going to do a gene therapy approach, you can put those in your cells and then, if something goes awry in the body, you can eliminate those cells, at least most of them, by adding an agent that will attack the new gene you put in, so that's there.* |
|  | 03 | Biomedical researcher | *So for modification of B-cells, the way people are thinking about that now is that they would have what's called a kill switch on the modification so that if they want to turn it on and off, they can. There's a lot of clever ways to do that... I do think you also need to pay attention to safety from the standpoint of having maybe a kill switch or a way to turn it off.* |
|  | 04 | Biomedical researcher | *You can put in these so called, suicide mechanisms. They're being used more and more in CAR T. But in CAR T, they're really being used to modulate the dose of the CAR T, so not to really eliminate them all together. Do we really have a situation where we can… actually modulate the dose post-infusion? Not really. You can treat it like any other lymphoproliferative disease if that's what occurs. Because the T-cells are still normal, they could be suppressed with steroids… It's good in many ways to try to develop these safety systems for shutting off interventions. I don't see any right now that I think really work… We have lots of on and off switches, and it's a false security to believe that they're really on or really off. So, I'm concerned about the ones that are being developed for clinical use because of this false security issue.* |
|  | 06 | Biomedical researcher | *Whether it's for gene edited cells or a CAR T-cell, one thing that we can do is introduce what's referred to as a suicide switch, and that is basically to program those cells so that, if we put some small molecule that is totally fine for an unmodified cell but will kill the modified cells, we can turn those cells off… By the same token, there's also ways that those modified cells could stay off. And then we add some sort of small molecule, it turns them on. So, there'd be so-called inducible systems, and it's another layer of moving parts, and another complication, but I think in a lot of diseases, not just HIV, that would be one way that we could control how long they work.* |
|  | 09 | Biomedical researcher | *Often these suicide therapies actually work before you want them to and, so, you actually end up killing all your cells before you actually do the trial.* |
|  | 11 | Biomedical researcher | *The kill switch. If you have a genetic modification where you have expression of a therapeutic, I think it's important that you have some way that you can shut that off for safety reasons in case something bad happens… I think you have to have the kill switch built in hoping that you're not going to need it and having data support that you're not going to need it, but just so that you have it there as a precaution.* |
|  | 17 | Biomedical researcher | *You could engineer in a safety switch, and a lot of studies are doing that now. There's genetic approaches that you can take to ensure that your product is safe, but it's going to come with some additional probably other risks as well, but you could do maybe a suicide gene if you really needed to control the duration or the timing.* |
|  | 19 | Biomedical researcher | *If you have a gene editing procedure that occurs, it needs to be quenchable, it needs to be easily controllable. That's a real critical thing. It needs to have a very effective on/off switch. That's really critical… Some of the gene editing and cell therapy stuff that we would do, that really isn't a concern because it would be a one-time thing. With the ex-vivo gene editing to get rid [of] core receptors, you could administer a mature Cas9 enzyme or protein into those cells to do the editing work, it does its work once, and that's it. It's a done deal. It gets degraded. It won't even be around to [do] any of the work anymore.* |
|  | 19 | Biomedical researcher | *There're like molecular switches you can use. Where you can administer a protein that would just shut something down, and you could just inject something into a patient's arm that would just deactivate the entire system.* |
| Need for long-term follow-up of CGT trial participants | 03 | Biomedical researcher | *I think another really important thing that has to happen is long term follow-up with people, right? You can't just do it for one year and then say it's all good. You have to be very careful with respect to how that's done in a follow-up.* |
|  | 11 | Biomedical researcher | *With gene therapy type approaches…, you have to follow individuals for a long period of time because, if it's something like a cancer risk, that's something that won't emerge for several years. So, that becomes a big challenge.* |
| Ultimately want durability of CGT to confer durable HIV control off ART | 07 | Biomedical researcher | *We're all of a mindset that we want durability. The only place we're willing to compromise on that durability is if it's the least invasive to the patient in terms of how the intervention is administered.* |
| Immune overreactions matter significantly | 06 | Biomedical researcher | *I think the immune overreaction matters a lot, even though the CAR T cells didn't really have much toxicity… That's something that we need to follow up on and really dig into as closely as possible.* |
|  | 07 | Biomedical researcher | *It's something that can be highly acute and also lead to death. It's something that could be chronic, requiring a lifetime of intervention… And then you haven't really... again, you've just sacrificed the need for antiretroviral therapy every day in exchange for long-term steroid use or something like that to control GvHD.* |
| More of an issue with CAR T-cells | 02 | Biomedical researcher | *Those could certainly be an issue with chimeric antigen receptor approaches because those are designed to turn on an immune response and, if you get an overwhelming immune response, you could get cytokine storm.* |
| Grading systems for cytokine release storms | 04 | Biomedical researcher | *But overall, we conduct training of physicians in sites because they may be ID [infectious diseases] people, they may not be familiar with cytokine response syndrome, they may not be familiar with ICANS which is the neurotoxicity grading syndromes for cell therapies.* |
| Need to test for antigenicity and immunogenicity | 19 | Biomedical researcher | *We're going to have to test very rigorously antigenicity or the immunogenicity of the delivery approaches.* |
| Pharmacological approaches to treat cytokine release storms | 02 | Biomedical researcher | *There's also ways that you can pharmacologically reduce inflammation. So, there's anti-inflammatories [and] there's biologics that will take out certain cytokines or block cytokine receptors. So, there are ways one can counter cytokine storm.* |
|  | 06 | Biomedical researcher | *It’s actually been very interesting that the types of immune activation that have been noted in COVID-19 patients are very similar to what is happening for CAR T-cells. So, there are antibodies that will neutralize some of the inflammatory molecules, like interleukin 6, or IL 6. There's an antibody called tocilizumab that neutralizes IL 6, and that's a good way to turn CAR T..., not turn CAR T-cells off so much, but turn off their immune activating response… Steroids, such as dexamethasone, are another good way of doing that, and, yeah, it's just been very interesting that that's how we can turn off these CAR T-cells, but it's turning out that COVID might be doing the same thing. So, we're also learning a lot just in these current times for how to do that.* |
|  | 10 | Biomedical researcher | *Adverse immune responses are absolutely monitored for, and you have people standing by with syringes full of anti-interleukin-6 and other mitigating effects so that, if you see that, you can stop it… Let's just make sure that we don't become so arrogant that we think that this is all going to go the way we expect [and] that we remain vigilant for these unexpected and very serious consequences of us trying to manipulate the immune system.* |
|  | 17 | Biomedical researcher | *CRS really comes about due to high antigen levels. The good thing about HIV that you can't say about cancer is that you can actually put people back on their ART… This is a way to bring down the viral antigen and so, therefore, you can reduce the chances of CRS. But then there's also again, and we have this written in our clinical protocol, other therapeutics that you can take to control CRS. So like Tocilizumab, corticosteroids.* |
| Active consultation with oncologists and infrastructure in place for dealing with cytokine release storms | 04 | Biomedical researcher | *Now, when you're doing CAR T, you're working with clinicians, with oncologists. Oncologists are used to poisoning people all the time, they think nothing of it. So, something like cyclophosphamide, a pretty dangerous compound, they don't hesitate to use. And when we come in now, in an infectious disease context, and we propose to use cyclophosphamide for conditioning, even though we're about one fourth the dose that they give for oncology, this raises all kinds of red flags.* |
|  | 17 | Biomedical researcher | *To be honest for clinical trials, you need to have an infrastructure in place that maybe contains some people from the cancer field that can help you if you were to have a CRS event to be able to mitigate this, get the right therapies, to have the right healthcare infrastructure to treat participants, if they experience such things.* |
